# Supplementary material for: Universal shape and pressure inside bubbles appearing in van der Waals heterostructures
Source: Nat Commun. 2016 Aug 25;7:12587. doi: 10.1038/ncomms12587 (PMC5007416; doi:10.1038/ncomms12587)
Supplement: Supplementary Information — Supplementary Figures 1-3 and Supplementary Notes 1-4 [file ncomms12587-s1.pdf]

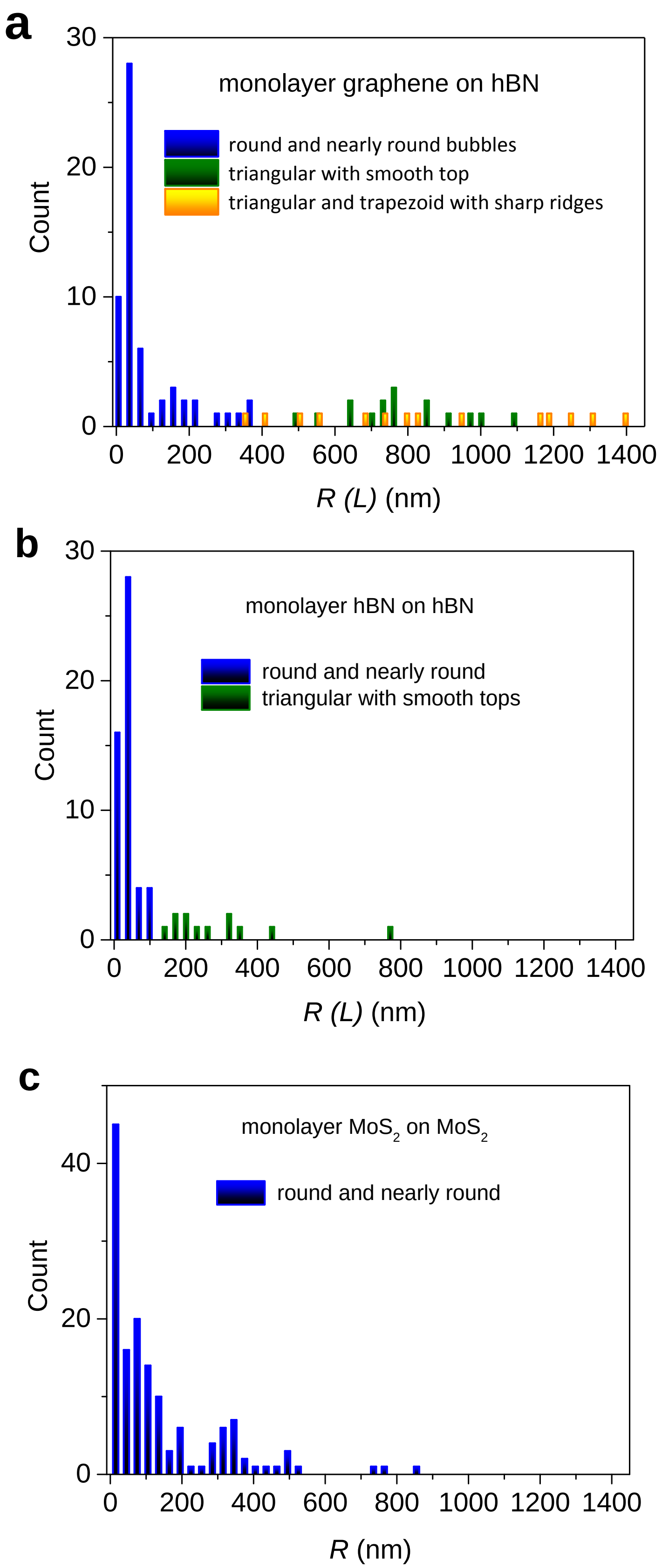

Supplementary Figure 1. Frequency of occurrence for bubbles of different shapes. Shown are histograms of bubble sizes corresponding to different shapes. **(a)** monolayer graphene on an hBN substrate ; data collected from an area of 1200 μm^2. **(b)** monolayer hBN on an hBN substrate, data collected from an area of 120 μm^2. **(c)** monolayer MoS_2 on an MoS_2 substrate, data collected from an area of 400 μm^2.

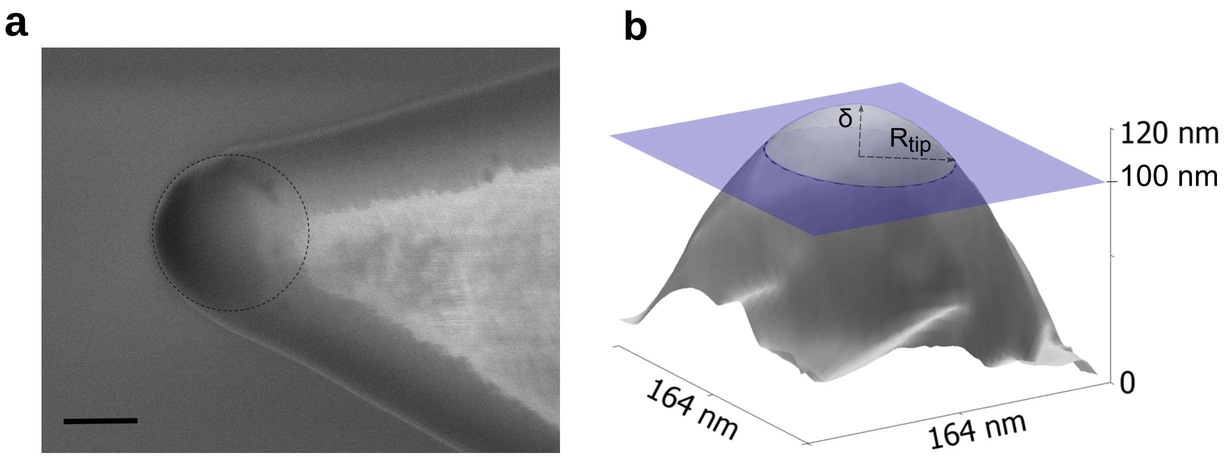

Supplementary Figure 2. **AFM tips used in indentation experiments. (a)** Scanning electron microscope (SEM) image of one of the AFM tips. The dashed circle emphasises its spherical shape. **(b)** Three-dimensional image of another AFM tip obtained using the Bruker calibration procedure (see Methods). The plane section indicates the maximum range of indentation depths corresponding to applicability of our numerical fitting procedure.

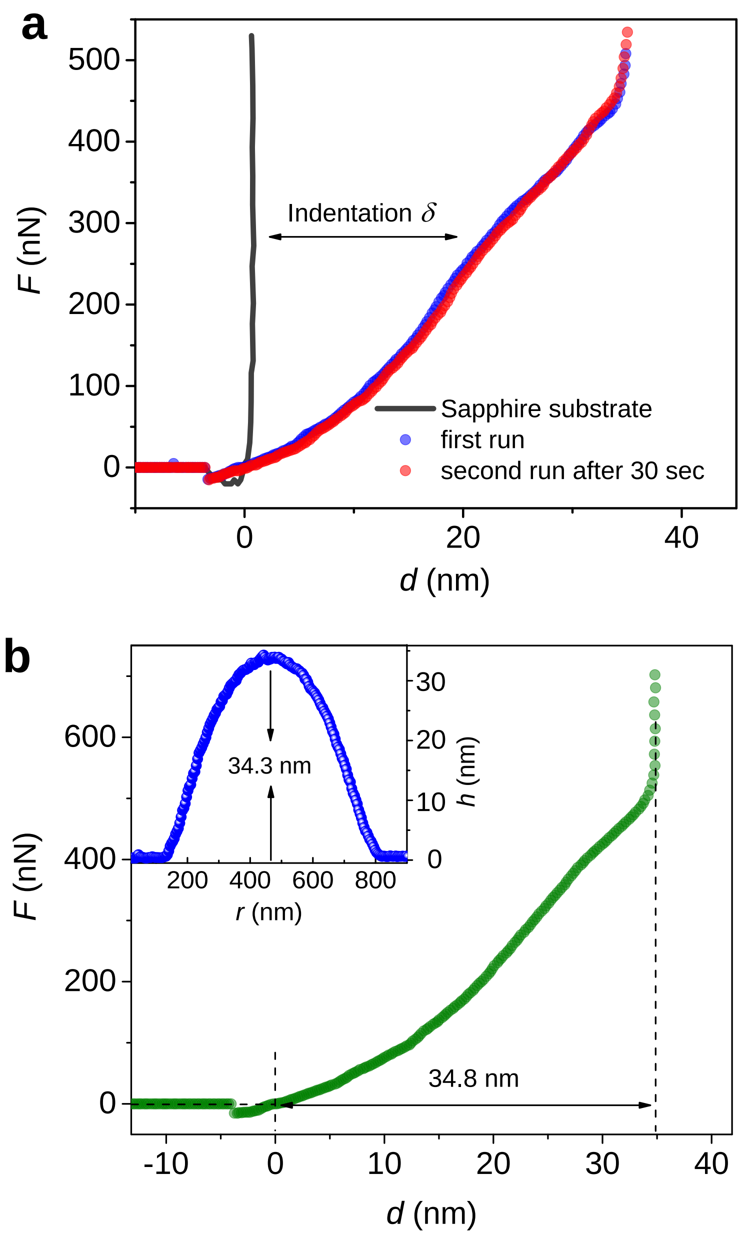

Supplementary Figure 3. Reproducibility of force-displacement curves and definition of zero indentation. (a) Two force-displacement (FDC) curves taken on the same graphene bubble (R =317 nm). Black line shows an FDC taken with the same AFM tip on a sapphire substrate. **(b)** Example of an FDC obtained by full-height indentation of a graphene bubble (see Methods); the inset shows a profile of the same bubble obtained in the scanning mode prior to indentation. Crossing dashed lines in the main panel indicate $\delta = 0$ (zero indentation); the vertical part of the FDC corresponds to indentation of the substrate (i.e., d reaches the height of the bubble, $h_{\max}$). The distance travelled by the AFM tip until it reaches the substrate, $d = 34.8$ nm is in excellent agreement with the measured bubble's height $h_{\max} = 34.3$ nm.

SUPPLEMENTARY NOTE 1. ELASTIC ENERGY.

The elastic energy of one bubble, defined by a profile $h(\vec{\mathbf{r}})$ is

$$E_{\mathrm{el}} = \frac{\mu(\lambda+\mu)}{2(\lambda+2\mu)} \frac{1}{4\pi^2} \int d^2\vec{\mathbf{q}} \left| \sum_{i,j} \left(1 - \frac{q_i q_j}{|\vec{\mathbf{q}}|^2}\right) f_{i,j}(\vec{\mathbf{q}}) \right|^2 \tag{S.1}$$

where $i, j = x, y$ and

$$f_{ij}(\vec{\mathbf{q}}) = \int d^2\vec{\mathbf{r}} e^{i\vec{\mathbf{q}}\vec{\mathbf{r}}} \partial_i h(\vec{\mathbf{r}}) \partial_j h(\vec{\mathbf{r}}) \tag{S.2}$$

For an isotropic bubble, $h(\vec{r}) = h(r)$, where $r = |\vec{\mathbf{r}}|$, Supplementary Equation (S.1) becomes

$$E_{\mathrm{el}} = \frac{\pi}{8} \frac{\mu(\lambda+\mu)}{\lambda+2\mu} \times \int_0^\infty dq q \left| \int_0^\infty dr r \left[J_0(qr) - J_2(qr)\right] (\partial_r h)^2 \right|^2 \tag{S.3}$$

where $J_0(u)$ and $J_2(u)$ are Bessel functions.

In the presence of an isotropic strain, $\epsilon_{xx} = \epsilon_{yy} = \epsilon/2$, the bubble acquires an additional energy,

$$E_{\mathrm{str}} = \frac{\epsilon\mu(\lambda+\mu)}{\lambda+2\mu} \lim_{\vec{\mathbf{q}}\to 0} \sum_{i,j} \left(1 - \frac{q_i q_j}{|\vec{\mathbf{q}}|^2}\right) f_{i,j}(\vec{\mathbf{q}}) \tag{S.4}$$

where we have used the relation $\epsilon(\vec{\mathbf{q}}) = 4\pi^2 \epsilon \delta^2(\vec{\mathbf{q}})$. For an isotropic height profile, $h(r)$, Supplementary Equation (S.1) reduces to

$$E_{\mathrm{str}} = \frac{\pi\epsilon\mu(\lambda+\mu)}{2(\lambda+2\mu)} \int_0^\infty dr r \left[\partial_r h(r)\right]^2 \tag{S.5}$$

Alternatively, for an isotropic height profile, $h(r)$, one can write the elastic energy as

$$E_{\mathrm{el}} = 2\pi \frac{\lambda}{2} \int_0^\infty dr r \left[\partial_r u_r + \frac{u_r}{r} + \frac{(\partial_r h)^2}{2}\right]^2 + 2\pi\mu \int_0^\infty dr r \left[\left(\partial_r u_r + \frac{(\partial_r h)^2}{2}\right)^2 + \frac{u_r^2}{r^2}\right] \tag{S.6}$$

The displacement u_r can be obtained from

$$-(\lambda+2\mu)\left(\partial_r^2 u_r + \frac{\partial_r u_r}{r} - \frac{u_r}{r^2}\right) = (\lambda+2\mu)\left(\partial_r^2 h\right)\left(\partial_r h\right) + \frac{\mu}{r}\left(\partial_r h\right)^2 \tag{S.7}$$

SUPPLEMENTARY NOTE 2. INFLUENCE OF TENSILE STRAINS.

Tensile strains, $\epsilon > 0$, modify the aspect ratio of the bubbles, as shown in Supplementary Equation (S.7) in the main text. The changes significant when the term proportional to ϵ becomes a significant fraction of the total elastic energy. By comparing these contributions in Supplementary Equation (S.7), we find a threshold strain, ϵ_{th}

$$\epsilon_{\mathrm{th}} \approx \frac{c_1}{c_2}\left(\frac{h_{\mathrm{max}}}{R}\right)^2 = \frac{c_1}{c_2}\left(\frac{\pi\gamma}{5Y}\right)^{1/2} \tag{S.8}$$

Using $\gamma \approx 0.015$ eV, and the Young modulii for graphene and MoS_2 we find, for graphene, $\epsilon_{\mathrm{th}} \approx 1.4\%$, and for MoS_2, $\epsilon_{\mathrm{th}} \approx 1.9\%$. For larger tensile strains,the aspect ratio tends to

$$\frac{h_{\mathrm{max}}}{R} \approx \sqrt{\frac{\pi\gamma}{2c_2\epsilon Y}} \tag{S.9}$$

The bubbles become shorter in the presence of a tensile strain. The dependence of the pressure on the height of the bubble, Supplementary Equation (S.18) in the main text is not significantly modified, and we find

$$P \approx \frac{\pi\gamma}{c_V h_{\mathrm{max}}} \tag{S.10}$$

SUPPLEMENTARY NOTE 3. INFLUENCE OF COMPRESSIVE STRAINS.

In the presence of compressive strains, $\epsilon < 0$, the aspect ratio $h_{\rm max}/R$, increases. A sufficiently high compressive strain will overcome the vdW interaction, and the graphene layer will delaminate, forming a bubble even in the absence of trapped material, or can even completely detach from the substrate. If we do not consider the internal energy of the material inside the bubble, a bubble formed spontaneously with radius R and height $h_{\rm max}$ has an energy,

$$E_{\rm tot} = c_1 Y \frac{h_{\rm max}^4}{R^2} - c_2 Y|\epsilon| h_{\rm max}^2 + \pi\gamma R^2 \tag{S.11}$$

Minimizing this expression with respect to $h_{\rm max}$, we find

$$\frac{h_{\rm max}^2}{R^2} = \frac{c_2|\epsilon|}{2c_1} \tag{S.12}$$

The total energy of the bubble, as function of R, is

$$E_{\rm tot} = -\frac{Y c_2^2 |\epsilon|^2}{4c_1} R^2 + \pi\gamma R^2. \tag{S.13}$$

The bubble will be stable and grow towards $R \to \infty$ if the compressive strain is such that

$$\frac{Y c_2^2 |\epsilon|^2}{4c_1} > \pi\gamma \tag{S.14}$$

The compressive strain required for the bubble to be unstable is

$$|\epsilon| \geq \sqrt{\frac{4c_1\pi\gamma}{Y c_2^2}} \tag{S.15}$$

These strains are 6.2% and 8.7% for graphene and MoS_2, respectively.

On the other hand, for sufficiently small strains, we can expand the solution of Supplementary Equation (S.8) in the main text, and we obtain

$$\frac{h_{\rm max}}{R} \approx \left(\frac{c_4\gamma}{5c_1 Y}\right)^{1/4} - \frac{c_2\epsilon}{10c_1}\sqrt{\frac{5c_1 Y}{\pi\gamma}}, \tag{S.16}$$

that is, for $\epsilon < 0$ (compressive strain) $h_{\rm max}/R$ will be greater than in the unstrained situation.

SUPPLEMENTARY NOTE 4. INTERACTION BETWEEN BUBBLES.

Bubbles can only interact when the in plane displacements relax to the height profile. We can extend eqs.(S.1) and (S.3) to the case of two non overlapping profiles, $h_1(\vec{\mathbf{r}})$ and $h_2(\vec{\mathbf{r}})$. Leaving out the self energy of each bubble, we obtain

$$E_{int} = \frac{\mu(\lambda+\mu)}{2(\lambda+2\mu)}\frac{1}{4\pi^2}\int d^2\vec{\mathbf{q}} e^{i\vec{\mathbf{q}}\vec{\mathbf{R}}} \times \left[\sum_{i,j}\left(1-\frac{q_i q_j}{|\vec{\mathbf{q}}|^2}\right) f_{i,j}^1(\vec{\mathbf{q}})\right]\left[\sum_{i,j}\left(1-\frac{q_i q_j}{|\vec{\mathbf{q}}|^2}\right) f_{i,j}^2(\vec{\mathbf{q}})\right] \tag{S.17}$$

where $\vec{\mathbf{R}}$ is the vector connecting the centers of the two bubbles, and $f_{i,j}^k(\vec{\mathbf{q}})$ is, as before, the Fourier transform of $\partial_i h_k(\vec{\mathbf{r}})\partial_j h_k(\vec{\mathbf{r}})$.

$$E_{\rm int}(R) = \frac{\pi}{8}\frac{\mu(\lambda+\mu)}{\lambda+2\mu}\int_0^\infty dq q J_0(qR) \times \left\{\int_0^\infty dr r \left[J_0(qr) - J_2(qr)\right](\partial_r h_1)^2 \times \int_0^\infty dr r \left[J_0(qr) - J_2(qr)\right](\partial_r h_2)^2\right\} \tag{S.18}$$

where $R = |\vec{\mathbf{R}}|$. A long distances $R \gg \ell$, we obtain

$$E_{\rm int}(R) \approx \frac{\alpha\pi}{8}\frac{\mu(\lambda+\mu)}{(\lambda+2\mu)R^2}\int_0^\infty drr\,(\partial_r h_1)^2\int_0^\infty drr\,(\partial_r h_2)^2 \tag{S.19}$$

where

$$\alpha = \lim_{q\to 0} r^2\int_0^\infty dqqJ_0(qr) \approx -1.11\times 10^{-6} \tag{S.20}$$

The interaction between bubbles is attractive, favouring their merger into bigger bubbles, as observed experimentally.
